# Adult-onset neuronal intranuclear inclusion disease related retinal degeneration: a Chinese case series

**DOI:** 10.3389/fmed.2024.1188193

**Published:** 2024-01-15

**Authors:** Chaoyi Feng, Qian Chen, Xinghua Luan, Ping Sun, Yuwen Cao, Jingying Wu, Shige Wang, Xinghuai Sun, Li Cao, Guohong Tian

**Affiliations:** ^1^Department of Ophthalmology, Eye Ear Nose and Throat Hospital of Fudan University, Shanghai, China; ^2^Department of Neurology, Shanghai Jiaotong University Affiliated Sixth People’s Hospital, Shanghai, China; ^3^State Key Laboratory of Medical Neurobiology and MOE Frontiers Center for Brain Science, Institutes of Brain Science, Fudan University, Shanghai, China; ^4^Shanghai Neurological Rare Disease Biobank and Precision Diagnostic Technical Service Platform, Shanghai, China

**Keywords:** neuronal intranuclear inclusion disease, *NOTCH2NLC* gene, retinal dystrophy, optical coherence tomography, fundus autofluorescence, pupilometer

## Abstract

**Purpose:**

To evaluate adult-onset neuronal intranuclear inclusion disease (NIID)-related retinopathy with guanine-guanine-cytosine repeat expansions in *NOTCH2NLC*.

**Materials and methods:**

Neuro-ophthalmic evaluations, including best-corrected visual acuity, slit-lamp biomicroscopy, intraocular pressure (IOP), ultrasound biomicroscopy, pupillometry, fundus photography, fundus autofluorescence (FAF), optical coherence tomography (OCT), Humphrey visual field, full-field electroretinography (ERG), and multifocal ERG (mf-ERG) were performed in patients with gene-proven NIID.

**Results:**

Nine patients (18 eyes) were evaluated, with a median age of 62 years (55–68) and only one man was included in our study. Six patients presented with decreased visual acuity or night blindness, whereas the other three were asymptomatic. The visual acuity was measured from 20/200 to 20/20. Miosis was present in eight patients, four of whom had ciliary process hypertrophy and pronation, and three of whom had shallow anterior chambers. Fundus photography, FAF, and OCT showed consistent structural abnormalities mainly started from peripapillary areas and localized in the outer layer of photoreceptors and inner ganglion cell layer. ERG and mf-ERG also revealed retinal dysfunction in the corresponding regions.

**Conclusion:**

Patients with NIID showed both structural and functional retinopathies which were unique and different from common cone-rod dystrophy or retinitis pigmentosa. Patients with miosis may have a potential risk of an angle-closure glaucoma attack. Neuro-ophthalmic evaluations is essential for evaluating patients with NIID, even without visual symptom.

## 1 Introduction

Neuronal intranuclear inclusion disease (NIID) is a rare progressive neurodegenerative disorder characterized by the deposition of eosinophilic hyaline intranuclear inclusions in neuronal and somatic cells, as well as various organs (1–9). The manifestation of neurological symptoms is highly variable, and the clinical subtypes can be divided into infantile, juvenile, and adult-onset forms according to the age of onset (10). Adult-onset NIID is observed in sporadic and familial cases (1, 10, 11). Skin biopsy and gene testing for repeat expansions of guanine-guanine-cytosine (GGC) in the *NOTCH2NLC* gene can differentiate between NIID and other neurodegenerative disorders (12–15).

Ocular dysfunction in patients with NIID has been reported in many studies, mainly focusing on pupillary abnormalities such as miosis, which were more frequently observed in sporadic cases (94.4%) than in familial cases (63.6%) (1). Tai et al. (16) evaluated 223 Chinese patients with NIID and found that only 14.3% had visual loss.

As early as 1991, patients with biopsy-proven adult-onset NIID demonstrated a marked reduction in the dark-adapted b-wave response (17). Recently, Omoto et al. described a patient with sporadic adult-onset NIID who developed retinal dystrophy and abnormal electroretinography (ERG) results, presenting with childhood-onset night blindness (3). Nakamura et al. (6) reported on the ocular characteristics of NIID disease-related retinopathy in seven Asian patients, revealing similar features, including rod-cone dysfunction with progressive retinal degeneration in the peripapillary and midperipheral regions. This was a detailed study on retinopathy including patients with GGC repeat expansion in NIID disease.

Meanwhile, ubiquitin-positive neuronal intranuclear inclusions, which were previously considered to be unique pathologic evidence of NIID, have also been detected in patients with fragile X-associated tremor or ataxia syndrome (18), frontotemporal lobar degeneration, and motor neuron disease or amyotrophic lateral sclerosis (19). Neuro-ophthalmic evaluations, such as OCT and ERG are very useful techniques to differentiate the spectrum disorders. Therefore, thoroughly neuro-ophthalmic examination in neurodegenerative disease is very helpful for differentiate diagnosis, due to some distinctive pattern of retinopathies.

However, pupil light reflexes, structural and functional retinal changes in NIID have not been investigated in detail. In the present study, we utilized various techniques including pupillometry, fundus autofluorescence (FAF), optical coherence tomography (OCT), and electrophysiology to evaluate nine sporadic cases of adult-onset NIID with GGC repeat expansions in *NOTCH2NLC*.

## 2 Materials and methods

### 2.1 Participants

The study participants were patients diagnosed with adult-onset NIID by neurologists at the Shanghai Jiaotong University Affiliated Sixth People’s Hospital between July 2020 and March 2021, and then referred to the Department of Ophthalmology, Eye, Ear, Nose, and Throat Hospital of Fudan University to undergo screening for visual involvement.

All patients with NIID were diagnosed based on their clinical symptoms, brain magnetic resonance imaging, skin biopsy showing eosinophilic and ubiquitin-positive intranuclear inclusions, and gene testing confirming GGC repeat expansions in the *NOTCH2NLC* gene. The study protocol adhered to the tenets of the Declaration of Helsinki and was approved by the local ethics committee. Written informed consent was obtained from each participant after explaining the nature and purpose of the study.

### 2.2 Clinical investigations

Demographic data, including gender, age, and visual symptoms, were recorded. A detailed medical history was obtained from the patients. Findings on routine ophthalmologic examinations in the form of best-corrected visual acuity were recorded and converted to an early treatment diabetic retinopathy study letter score. Intraocular pressure (IOP) and depth of the anterior chamber were also evaluated by ultrasound biomicroscopy (UBM).

Pupillary sizes and light reflexes were tested using a pupilometer (NeurOptics DP2000; NeurOptics Inc., Irvine, CA, United States), a binocular system that stimulates the eyes directly, consensually, or bilaterally simultaneously and features automatic tracking and pupil detection. Spectral-domain OCT (SD-OCT) scans were performed using the Heidelberg Spectralis (Heidelberg Engineering Inc., Heidelberg, Germany). The macular ganglion cell-inner plexiform layer (GCIPL) and peripapillary retinal nerve fiber layer (RNFL) were measured using the Cirrus HD-OCT Model 4000 (Carl Zeiss Meditec AG, Jena, Germany). Other ophthalmic examinations included the Humphrey visual field (HVF) (Carl Zeiss Meditec AG) examination, ultrasound biomicroscopy (UBM; SUOER), fundus photography (Optos 200Tx), FAF (Optos 200Tx; Optos Plc, Dunfermline, Scotland), ERG (UTAS visual electrodiagnostic test system; LKC Technologies, Gaithersburg, MD, United States), and mf-ERG (VERIS; Electro-Diagnostic Imaging Inc., CA, United States).

## 3 Results

### 3.1 Demographics and ophthalmic tests

Nine patients diagnosed with gene proven NIID were evaluated. The median age of the patients was 62 years (range, 55–68). Among them, eight patients (88.9%) were women. Six patients had ocular symptoms such as blurred vision, decreased visual acuity, and night blindness. Furthermore, slit-lamp examination found six patients had mild to moderate age-related cataracts, and one patient had stellar vitreous degeneration. No patients had a high IOP level (more than 20 mm Hg) and UBM revealed that four patients with miosis had ciliary process hypertrophy and pronation, with shallow anterior chambers (anterior chamber deep less than 2.3 mm, Supplementary Content 1). One patient who underwent intraocular lens implantation had normal anterior chamber depth and angle, however, a hypertrophic ciliary process was still present. Detailed ophthalmic characteristics are shown in Table 1.

**Table 1:** Demographics and clinical characteristics of the nine patients with NIID.

No.	Age (y)	Gender	Age of neurological (visual) symptoms onset (y)	Number of GGC repeat expansions	Nervous system symptoms	Visual symptoms	BCVA		Anterior chamber	Ciliary process	Other ocular condition (OU)
							OD	OS			
1	66	F	56	70	Syncope Autonomic dysfunction	None	20/20	20/20	Normal	Normal	Intraocular lens
2	59	F	54(56)	85	Cognitive impairment Tremor Headache	Decreased visual acuity	20/40	20/60	Shallow	Hypertrophy and pronation	Moderate cataract
3	58	F	56	100	Autonomic dysfunction Vomiting Dizziness	None	20/20	20/20	Normal	Normal	None
4	67	F	60(58)	120	Cognitive impairment Movement disorder	Blurred vision	20/25	20/25	Shallow	Hypertrophy and pronation	Mild cataract, stellar vitreous degeneration
5	66	F	63	125	Cognitive impairment Movement disorder	None	20/20	20/25	Shallow	Hypertrophy and pronation	Mild cataract Iridectomy
6	55	F	50(52)	120	Movement disorder Behavior and psychiatric symptoms	Decreased visual acuity	20/50	20/50	Normal	Normal	None
7	68	F	66(60)	122	Visual decrease Cognitive impairment autonomic dysfunction	Night blindness	20/30	20/30	Normal	Normal	Mild cataract
8	64	F	60(58)	100	Behavior and psychiatric symptoms	Decreased visual acuity	20/30	20/30	Normal	Normal	Mild cataract
9	55	M	52(49)	140	Autonomic dysfunction Seizure Visual loss	Night blindness	20/100	20/400	Normal	Normal	Mild cataract

BCVA, best corrected visual acuity; F, female; GGC, guanine-guanine-cytosine; M, male; OD, right eye; OS, left eye; OU, both eyes; NIID, neuronal intranuclear inclusion disease.

### 3.2 Pupillary sizes and light reflexes

**Figure 1:**
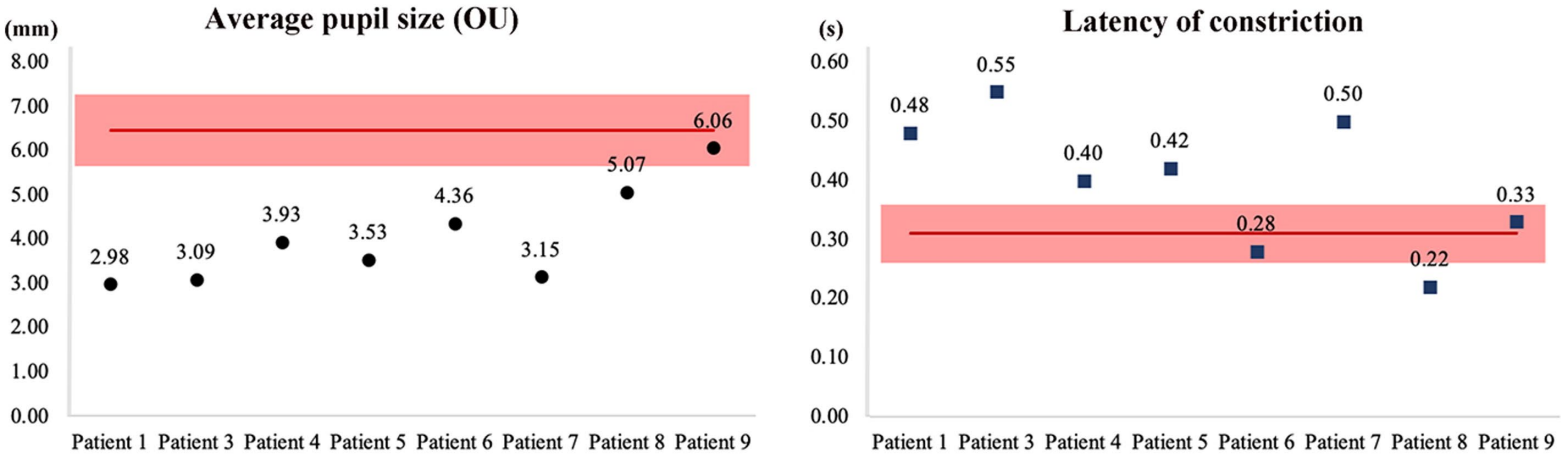
Pupil sizes and light reflexes in patients with NIID. The mean pupil sizes and latency of the pupillary light reflexes in the normal population (*red lines*), and their standard deviation (*red blocks*). Patient 2 was unable to complete the pupilometer tests. NIID, neuronal intranuclear inclusion disease.

Eight individuals (all except patient 9) presented with miosis. The patients had a mean mesopic pupil diameter of 4.02 mm. The pupillary light reflexes of six patients were sluggish and presented an increased latency of constriction. Among them, patient 2 was unable to complete the pupilometer test (Figure 1).

### 3.3 Structural retinal changes

**Figure 2:**
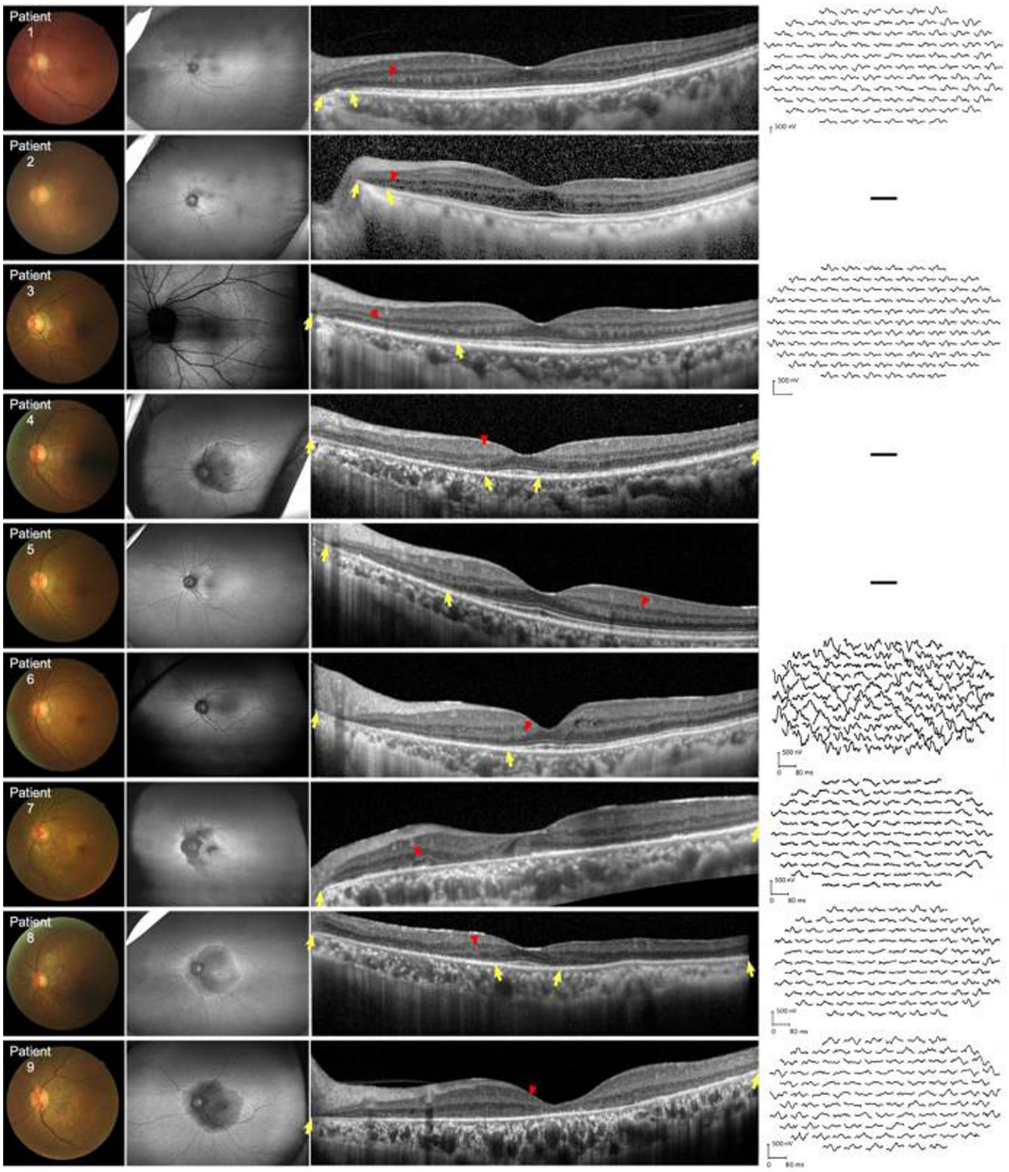
Fundus color, FAF, SD-OCT, and mf-ERG trace of nine patients with NIID. First left column: Fundus photographs show peripheral retinal pigmentary changes encroaching the fovea. Second left column: FAF images show constriction of the hyperautofluorescent ring corresponding to the border of EZ abnormalities. All patients had outer retinal dystrophy in the peripapillary regions. Patient 3 was assessed via Heidelberg OCT. In patients 4, 7, 8, and 9, dystrophy extended to the posterior poles which appeared as hypoautofluorescence. Third left column: horizontal SD-OCT cross-sections passing through the fovea for each patient showed EZ dystrophy (*yellow arrows*) and blurred IZ extending from the peripapillary regions to the posterior poles. Thinning of the outer nuclear layer can be seen in the corresponding regions. Hyperreflective fine dots (*red arrows*) scattered within the retina in all patients. Right column: mf-ERG of patients 1, 3, 6, 7, 8, and 9 revealed decreased b-wave amplitudes corresponding to the region of EZ and IZ abnormalities. EZ, ellipsoid zone; FAF, fundus autofluorescence; IZ, interdigitation zone; mf-ERG, multifocal full-field electroretinography; OCT, optical coherence tomography; SD-OCT, spectral-domain OCT.

**Figure 3:**
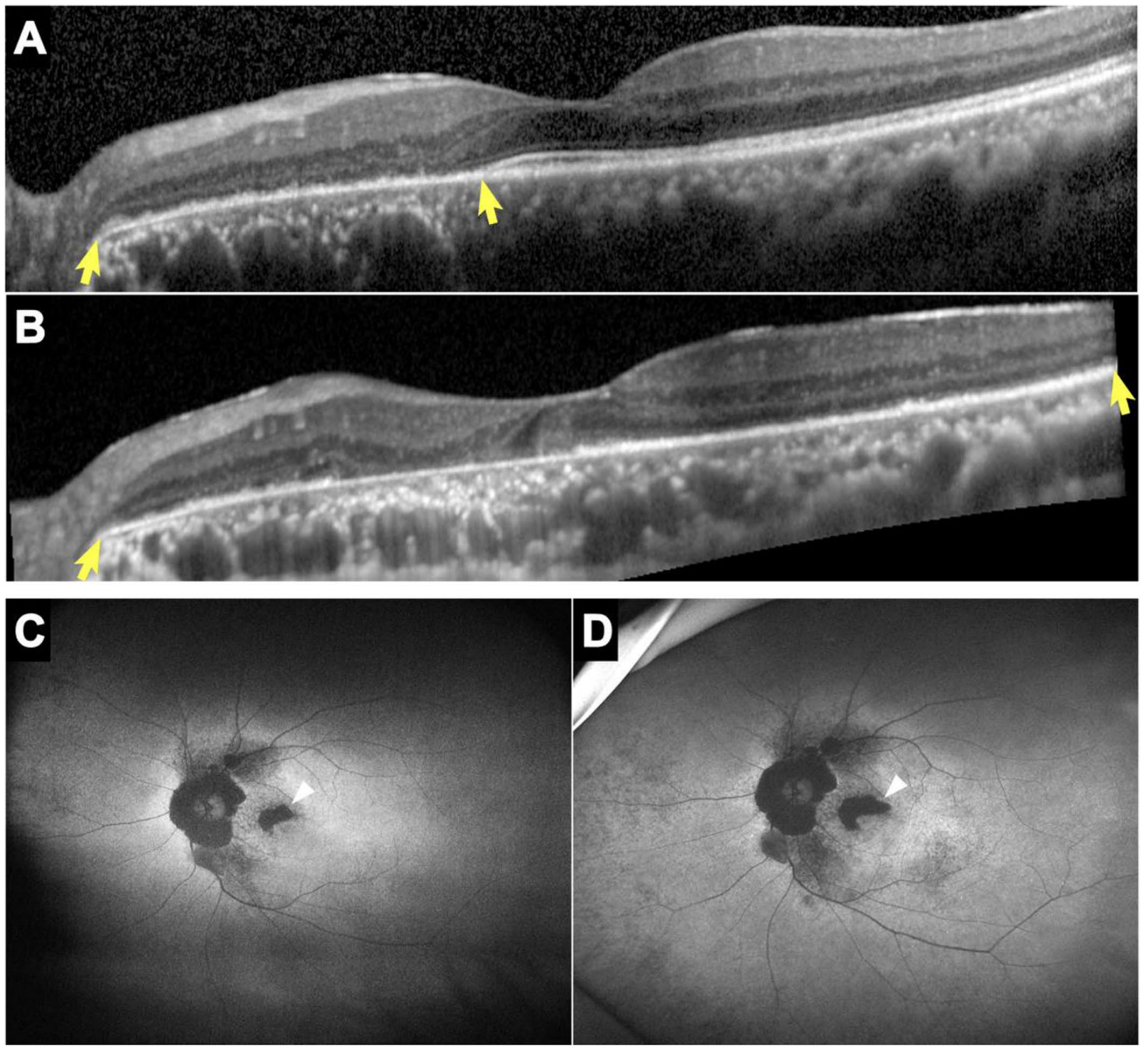
SD-OCT and FAF images of patient 7. Horizontal SD-OCT cross-sections passing through the fovea showed EZ dystrophy extending from the peripapillary regions to the entire posterior pole (*yellow arrows*) with loss of the IZ and outer nuclear layer (**A**: year 2014, **B**: year 2020). FAF showed hypoautofluorescent patches that enlarged noticeably over 2 years, particularly in the parafoveal regions (*white arrows*) (**C**: year 2018, **D**: year 2020). EZ, ellipsoid zone; FAF, fundus autofluorescence; IZ, interdigitation zone; SD-OCT, spectral-domain optical coherence tomography.

All patients showed structural retinal abnormalities in both eyes, including those without visual symptoms. Fundus photographs showed peripapillary and posterior pole area retinal pigmentary changes encroaching the fovea. Additionally, wide-field FAF showed that the patients had different degrees of hypoautofluorescent areas in the peripapillary regions or posterior pole areas, indicating outer retinal dystrophy. Among them, patients 4, 5, 7, 8, and 9 had larger photoreceptor dystrophy regions that extended to the posterior poles, and hyperautofluorescent rings surrounding the hypoautofluorescent regions, indicating the border of the lesion (Figure 2). SD-OCT revealed structural changes in the outer retina throughout the patients, such as an ellipsoid zone (EZ) thinning, blurred interdigitation zone (IZ), and thinning of the outer nuclear layer. Hyperreflective fine dots were scattered throughout several of the eye layers in each patient, as shown in Figure 2. The areas of EZ abnormality shown on SD-OCT corresponded to the hyperautofluorescent rings on the wide-field FAF. Interestingly, patient 7 presented with night blindness 6 years prior to the diagnosis of NIID. The follow-up SD-OCT revealed progressive outer retinal dystrophy showing disruption of the EZ in the peripapillary regions initially, followed by the whole layer structure disappearing. The outer retinal abnormalities manifested as hypoautofluorescent patches in the FAF within the peripapillary and parafoveal regions and dramatically enlarged during the 2 years period (Figure 3). The nerve fiber layer of the peripapillary optic nerve (RNFL) and GCIPL of macular thickness were measured in six patients showing a decline in the peripapillary RNFL thickness in patients 4, 7, and 8 (Supplementary Content 1). All six patients had reduced macular GCIPL thickness; however, patients 4, 7, 8, and 9 had a significantly thinner macular GCIPL.

### 3.4 Functional retinal changes

**Figure 4:**
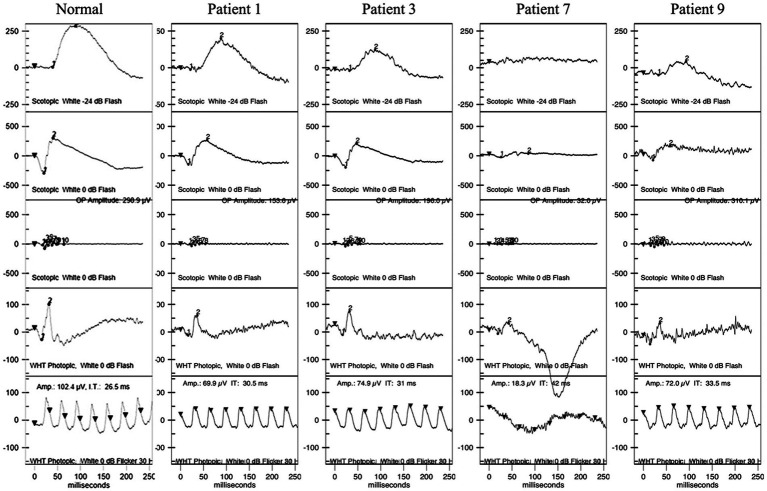
ERG of patients 1, 3, 7, and 9. Patient 1 had normal waves in dark adaptation. Patients 3, 7, and 9 had decreased amplitudes and increased latency of the b wave in dark adaptation with different degrees. All four patients had decreased amplitudes and increased latency in light adaptation. ERG, full-field electroretinography.

Five patients underwent HVF tests, revealing physiological blind spot enlargement, bilateral temporal field defects that did not respect the vertical meridian, and visual field constriction (Supplementary Content 1). Furthermore, ERG and mf-ERG were performed on six of the patients without shallow anterior chambers, cases in which it was safe to dilate the pupils. ERG showed abnormalities in both retinal dark adaptation and light adaptation, including decreased amplitudes and increased latencies in all six patients (Figure 4). mf-ERG revealed various degrees of diffusely and asymmetrically decreased b-wave amplitudes and increased latencies in the posterior pole areas, indicating photoreceptor dysfunction. These functional abnormalities corresponded to the structural disruptions identified on FAF and OCT examinations. Discussion.

## 4 Discussion

### 4.1 Key findings

In 2019, Chinese and Japanese scholars revealed the causative gene of NIID. Due to relatively rare data, the prevalence of NIID is unclear; however, it is known to be relatively high in Japan and China, where a large percentage of the cases are reported (1–3, 13, 15, 16, 20).

Autonomic dysfunction of the pupils is frequently seen in patients with NIID (1–3, 6, 10, 11, 17, 21). However, previous studies have not quantified pupil sizes and light reflexes using pupillometry, a method which can reveal more information regarding the condition. We observed that the mean pupil diameters in patients with NIID ranged from 3.09 to 6.06 mm, dramatically smaller than the normal diameter range of 5.63 to 7.27 mm (22). The average latency of the pupillary light reflexes was 0.31 ± 0.05 s (23). The rate of miosis in our study was 88.9% (8/9) and the pupillary light reflexes were sluggish, as reported previously (17, 24). The unique feature of pupil size and light response is very helpful to ophthalmologists in identifying parasympathetic dysfunction among patients with NIID, which is usually diagnosed by a neurologist. One-third of the patients had shallow anterior chambers; however, as the pupillary sphincter and ciliary muscle are both parasympathetically innervated, we hypothesized that autonomic dysfunction may lead to relative hyperactivity of the parasympathetic nerve, causing pupil constriction and excessive contraction of the ciliary muscle, thereby increasing the risk of an angle-closure glaucoma attack. Notably, patient 5 underwent an iridectomy after evaluation as there were signs of possible acute attack of the glaucoma. Our findings suggest that patient’s with very shallow anterior chambers should be evaluated for prophylactic treatment to ensure the best clinical outcomes.

OCT and visual electrophysiology techniques verified that retinopathies, especially involving the outer layer of photoreceptors, were the initial and main sites of involvement in adult-onset NIID with GGC repeat expansions in *NOTCH2NLC*, a finding consistent with previous reports (2, 3, 6). Based on the follow-up OCT data of patient 7, we hypothesized that retinal dystrophy began in the peripapillary region in the early-stage and progressed centrifugally toward the fovea areas.

In addition, thinning of the macular GCIPL was present in all patients who underwent measurement, even with mild outer retinal dysfunction, which has not been mentioned in previous reports. This suggests that ganglion cells may also be involved in NIID-related retinal lesions. It has been reported that neuronal intranuclear inclusion bodies have been found in retinal ganglion cells. Moreover, ganglion cells and retinal nerve fiber loss of myelinated axons of the optic nerve were observed as histopathological findings of monozygotic twins with NIID (21), providing evidence for the thinner macular GCIPL instances in our study. Additional evidence of RGC pathological lesions were the hyperreflective fine dots observed in ganglion cell layer as well as other layers in retina using SD-OCT. Little is known about the pathogenesis of retinal involvement in patients with NIID. Photoreceptor cells, ganglion cells and retinal nerve fiber are parts of the visual nervous system. Since NIID shows extensive neurologic involvement, it is hypothesized that retinopathy associated with NIID has a similar mechanism.

Modes of functional visual electrophysiology such as ERG and mf-ERG can be very helpful in differentiating between specific NIID-related retinopathy and well-known cone-rod dystrophy or retinitis pigmentosa, which are caused by different gene mutations. The pattern of retinopathies within the patients with NIID in our study could not be simply classified as cone-rod dystrophy or rod-cone dystrophy, especially as only six patients fulfilled ERG and the degree of the lesions were diverse. At early stage (without visual loss, case 1 and case 5), the lesion began from the peripapillary areas and gradually involving the macular, which the lesion is not centered by macular. At late stage, the lesions were very subtle and focus in cone dysfunction, develop all over the retina, which the pattern was as well as retinitis pigmentosa. In classic cone-rod dystrophy, the lesions are usually centered within the macular region and a bulls-eye-like sign can be seen under ophthalmoscopic examination. This developed pattern of lesions from posterior pole to peripheral retina is also different from typical rod-cone dystrophy. Therefore, we addressed the pattern of retinopathies in NIID is unique as displayed by the findings.

Due to the heterogenous features and phenotypes of NIID, it is very challenging to diagnose without biopsy or gene testing for GGC repeat expansions in the *NOTCH2NLC* among atypical patients. According to the recently large sample study, Tai et al. reported less than 15% of patients with NIID had symptoms of visual loss (16). According to our data, some of the retinopathies were subtle and started from the peripapillary areas rather than the macular region. Therefore, patients often may not present with visual symptoms unless at the late-stage of the disease. As the eye is a visualized window of the central nervous system, detecting early structural and functional deterioration changes in the retina via ophthalmic techniques is an effective and less invasive diagnostic method. We found that comprehensive ophthalmic examinations provided valuable information to aid a physician’s ability to evaluate the diagnosis and prognosis of patients with NIID.

### 4.2 Limitations

The present study was a small sample observational clinical study. To solve this issue we will continue to recruit patients with NIID spectra. Additionally, a prospective follow-up study is needed to provide the long-term results.

## 5 Conclusion

In conclusion, adult-onset NIID with GGC repeat expansions in *NOTCH2NLC* is associated with a unique pattern of retinopathies, mainly originating from the peripapillary areas and involving the outer layer of photoreceptors and inner ganglion cells layer. Additionally, miosis and sluggish pupillary reflexes are often accompanied by NIID. Our findings provide evidence that neuro-ophthalmic evaluations, especially OCT, is essential for evaluating patients with NIID, even without visual symptom.

## Data availability statement

The original contributions presented in the study are included in the article/Supplementary material, further inquiries can be directed to the corresponding authors.

## Ethics statement

The studies involving humans were approved by the Institutional Ethics Review Board of the Eye, Ear, Nose, and Throat Hospital of the Fudan University Shanghai. The studies were conducted in accordance with the local legislation and institutional requirements. The participants provided their written informed consent to participate in this study. Written informed consent was obtained from the individual(s) for the publication of any potentially identifiable images or data included in this article.

## Author contributions

GT and LC designed and conceptualized the study and revised the manuscript and discussions. CF, PS, QC, and YC was the major role in data acquisition. JW, XL, and SW interpreted the data. CF and XS performed statistical analysis. CF and QC drafted the manuscript for intellectual content. XS was supported by the grant. All authors contributed to the article and approved the submitted version.
