# Supplementary material for: Adult-onset neuronal intranuclear inclusion disease related retinal degeneration: a Chinese case series
Source: Front Med (Lausanne). 2024 Jan 15;11:1188193. doi: 10.3389/fmed.2024.1188193 (PMC10822994; doi:10.3389/fmed.2024.1188193)
Supplement: Supplementary file 1 [file Data_Sheet_1.DOCX]

| Patient | Gender | Age | Symptoms | BCVA  OD/OS | Pupil | Anterior Chamber | Other findings | Fundus | Opto | AF | VF | SD-OCT | RNFL | GCIPL | VEP | ff-ERG | mf-ERG | UBM |
| --- | --- | --- | --- | --- | --- | --- | --- | --- | --- | --- | --- | --- | --- | --- | --- | --- | --- | --- |
| 1 | Female | 66 | None | 0.9/0.9 | miosis | normal | IOL | 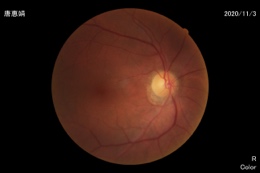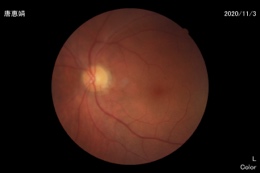 | 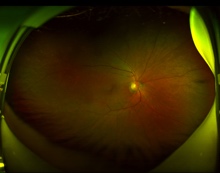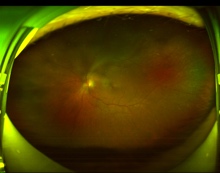 | 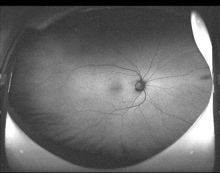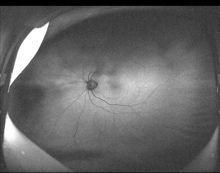 |  | 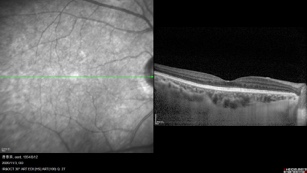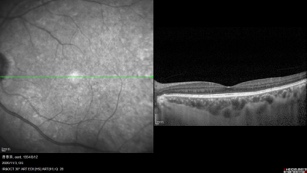 |  |  | - |  | 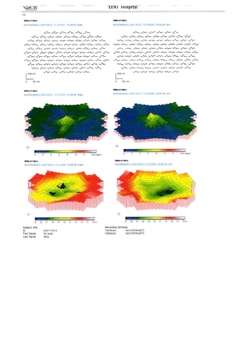 |  |
| 2 | Female | 59 | Decreased visual acuity | 0.5/0.3 | miosis | shallow | cataract | 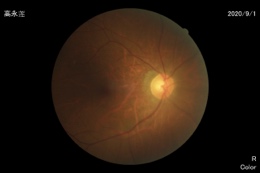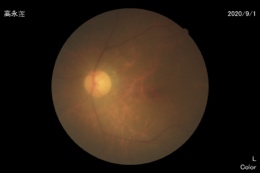 | 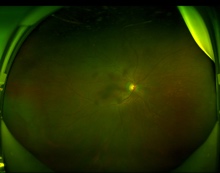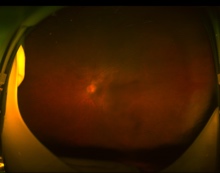 | 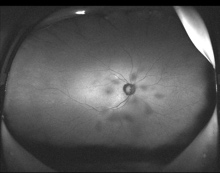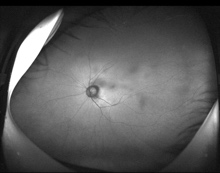 | - | 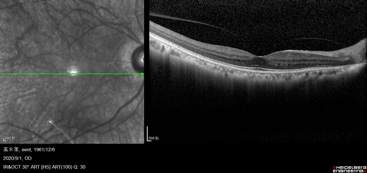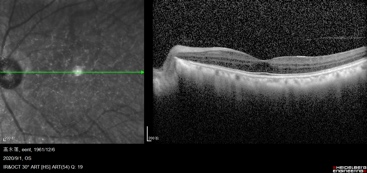 | - | - | - | shallow anterior chamber | shallow anterior chamber | - |
| 3 | Female | 58 | none | 1.0/1.0 | miosis | normal | - | 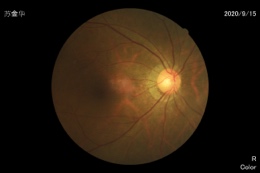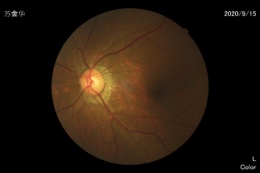 | - | 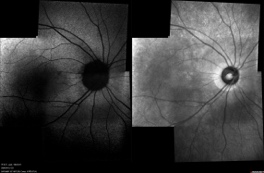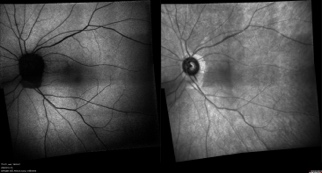 | - | 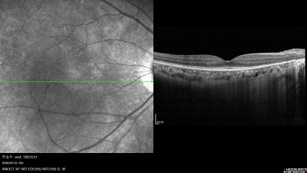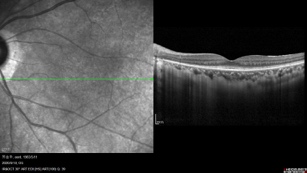 |  |  |  |  | 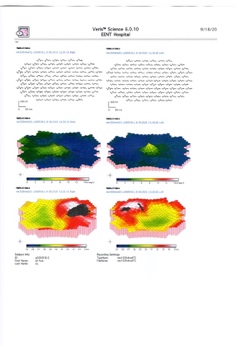 |  |
| 4 | Female | 67 | Blurred vision | 0.9/0.9 | miosis | shallow | cataract, Stellar vitreous degeneration | 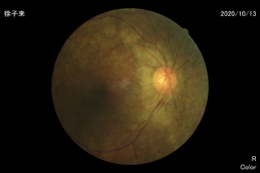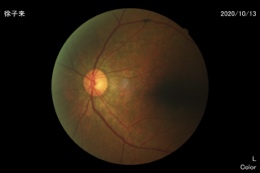 | 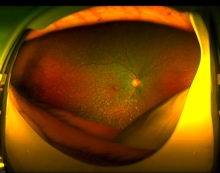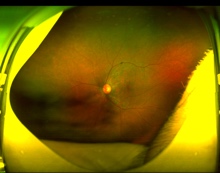 | 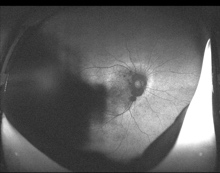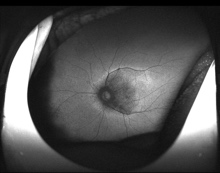 |  | 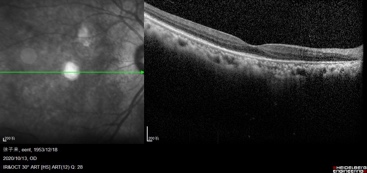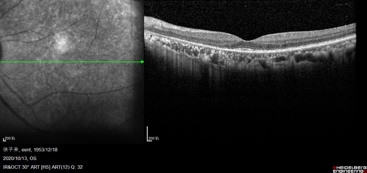 |  |  | - | shallow anterior chamber | shallow anterior chamber |  |
| 5 | Female | 66 | None | 1.0/0.9 | miosis | shallow | cataract | 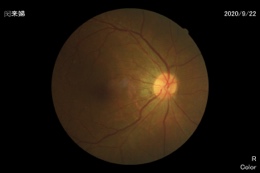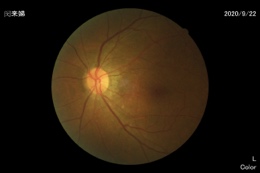 | 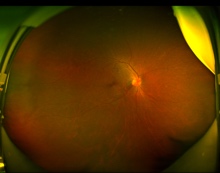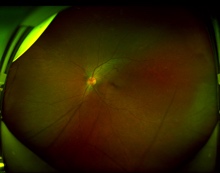 | 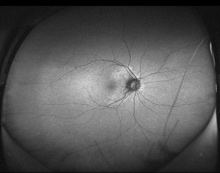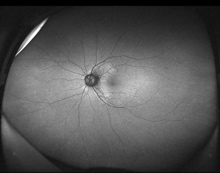 |  | 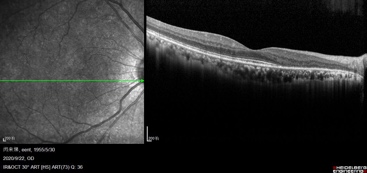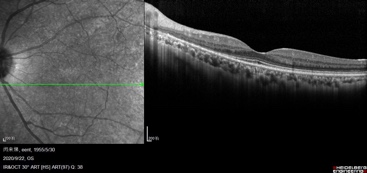 | - | - | - | shallow anterior chamber | shallow anterior chamber |  |
| 6 | Female | 55 | Decreased visual acuity, | 0.4/0.4 | normal | normal | - | 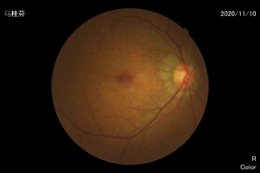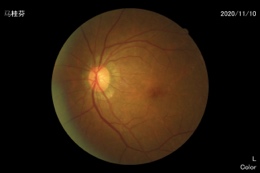 | 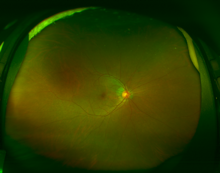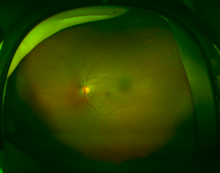 | 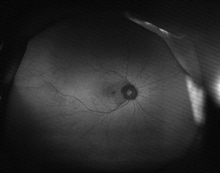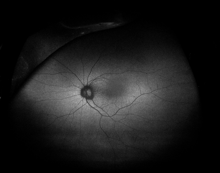 |  | 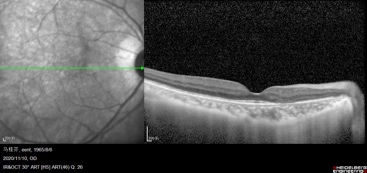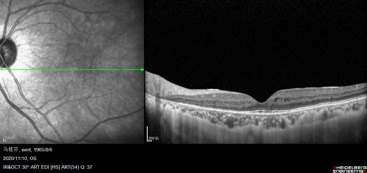 | 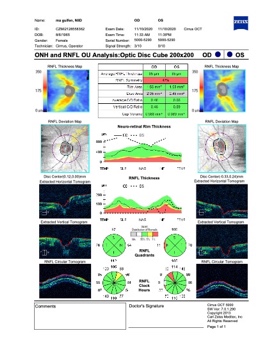 |  | - |  | 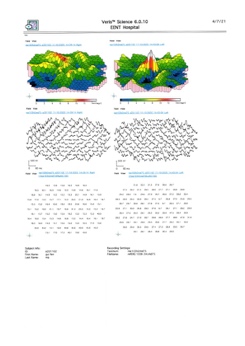 |  |
| 7-2014.11→2014 | Female | 62 | Decreased visual acuity, night blindness |  | miosis | normal | cataract | - | - | - | - | 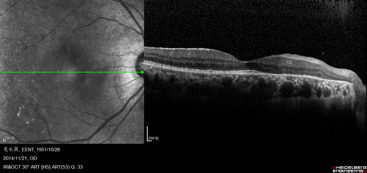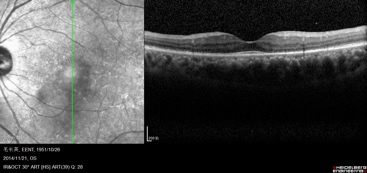 | - | - | - | - | - | - |
| 2019.3→2018 | Female | 67 |  |  |  |  | cataract |  | 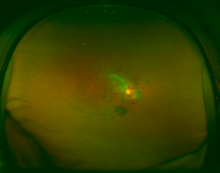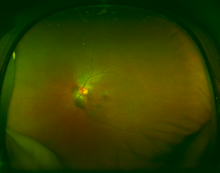 | 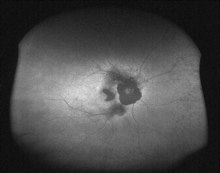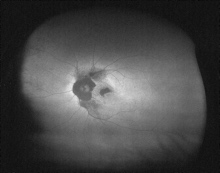 |  |  |  |  |  |  |  |  |
| 2020.10-2021.1→2020 | Female | 68 |  | 0.6/0.7 |  |  | cataract | 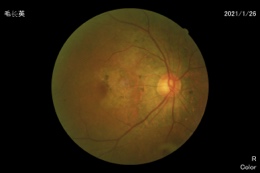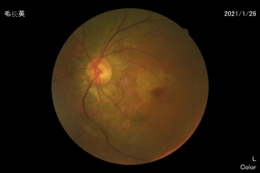 | 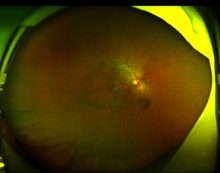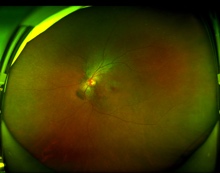 | 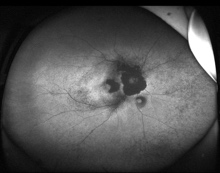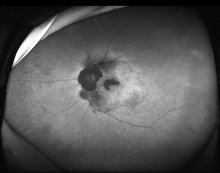 | 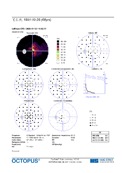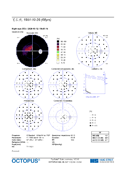 | 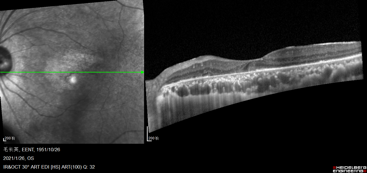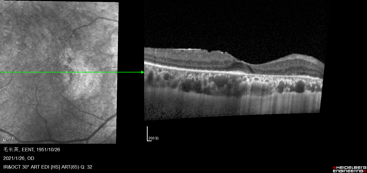 |  |  | - |  | 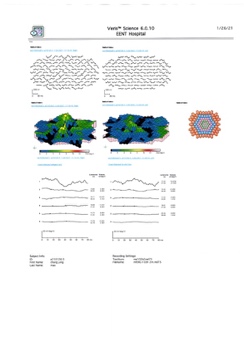 |  |
| 8 | Female | 64 | Decreased visual acuity | 0.6/0.6 | normal | normal | cataract | 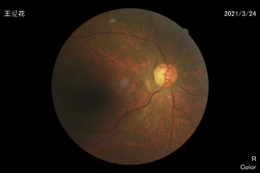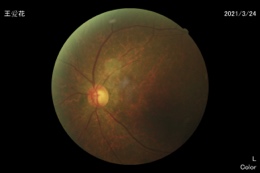 | 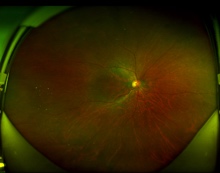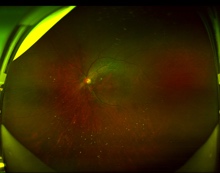 | 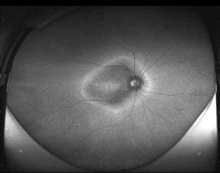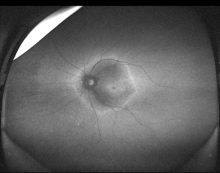 | 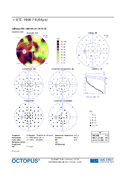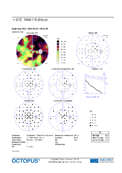 | 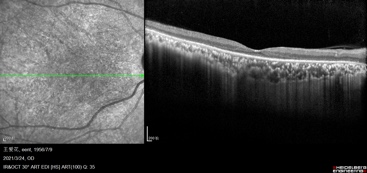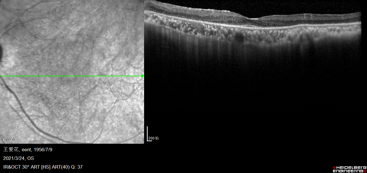 |  |  | - |  | 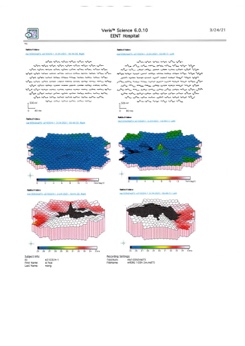 |  |
| 9 | Male | 55 | Decreased visual acuity | 0.2/0.05 | normal | normal | cataract | 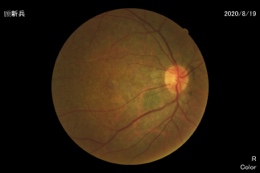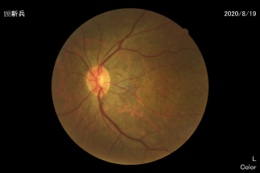 | 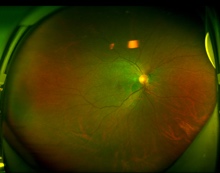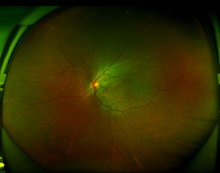 | 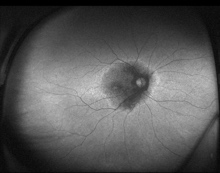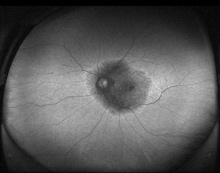 | 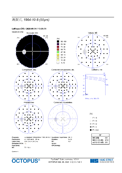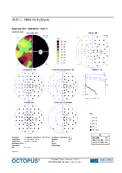 | 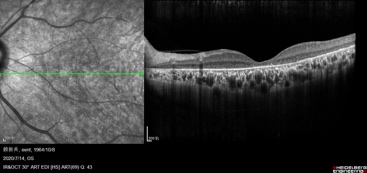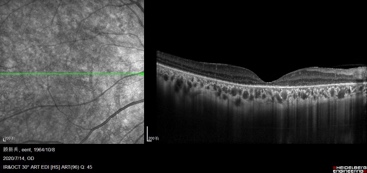 |  |  |  |  | 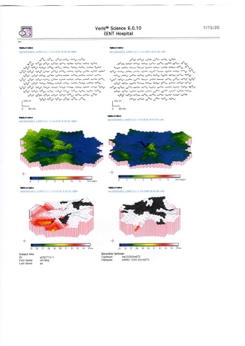 |  |
